# Permeabilization of the Blood-Brain Barrier via Mucosal Engrafting: Implications for Drug Delivery to the Brain

**DOI:** 10.1371/journal.pone.0061694

**Published:** 2013-04-24

**Authors:** Benjamin S. Bleier, Richie E. Kohman, Rachel E. Feldman, Shreshtha Ramanlal, Xue Han

**Affiliations:** 1 Department of Otology and Laryngology, Massachusetts Eye and Ear Infirmary, Harvard Medical School, Boston, Massachusetts, United States of America; 2 Department of Biomedical Engineering, Boston University, Boston, Massachusetts, United States of America; St. Jude Children’s Research Hospital, United States of America

## Abstract

Utilization of neuropharmaceuticals for central nervous system(CNS) disease is highly limited due to the blood-brain barrier(BBB) which restricts molecules larger than 500Da from reaching the CNS. The development of a reliable method to bypass the BBB would represent an enormous advance in neuropharmacology enabling the use of many potential disease modifying therapies. Previous attempts such as transcranial catheter implantation have proven to be temporary and associated with multiple complications. Here we describe a novel method of creating a semipermeable window in the BBB using purely autologous tissues to allow for high molecular weight(HMW) drug delivery to the CNS. This approach is inspired by recent advances in human endoscopic transnasal skull base surgical techniques and involves engrafting semipermeable nasal mucosa within a surgical defect in the BBB. The mucosal graft thereby creates a permanent transmucosal conduit for drugs to access the CNS. The main objective of this study was to develop a murine model of this technique and use it to evaluate transmucosal permeability for the purpose of direct drug delivery to the brain. Using this model we demonstrate that mucosal grafts allow for the transport of molecules up to 500 kDa directly to the brain in both a time and molecular weight dependent fashion. Markers up to 40 kDa were found within the striatum suggesting a potential role for this technique in the treatment of Parkinson’s disease. This proof of principle study demonstrates that mucosal engrafting represents the first permanent and stable method of bypassing the BBB thereby providing a pathway for HMW therapeutics directly into the CNS.

## Introduction

Neurologic disorders affect more than 20 million patients in the US alone and account for over $400 billion in annual expenditure for both their treatment and chronic care [1], [2]. The paucity of effective neurodegenerative therapies is directly attributable to the presence of the blood-brain barrier(BBB) and blood-cerebrospinal fluid barrier(BCSFB). These barriers are composed of densely packed cells with copious intercellular tight junctions(TJ), active efflux pumps, and a trilamellar basement membrane which prevent the absorption of polar or high molecular weight(HMW) molecules larger than 500Da [3]. This results in restriction of up to 98% of all potential neuropharmaceutical agents from reaching the central nervous system(CNS) [3]. Consequently, known charged or macromolecular therapies which may be capable of preventing or even reversing certain neurologic diseases are rendered clinically ineffective due to their inability to cross the BBB.

The limitations of the BBB have catalyzed a considerable research effort into ways to circumvent this barrier. Currently described experimental methods such as transcranial catheter placement [4] and the use of protein conjugates [1] may be invasive or require extensive drug manipulation to optimize trans-BBB transport rendering them impractical, morbid, and expensive to scale up for widespread clinical use. Furthermore even if the BBB could be successfully overcome, the majority of these strategies rely on a peripheral delivery route leading to potential systemic toxicity and pulsatile CNS delivery [2].

The evident value of a direct pathway capable of continuous delivery of polar and macromolecular therapies has led to a large body of work seeking to exploit the olfactory mucosa for this purpose. Trans-olfactory CNS uptake of nerve growth factor(NGF, 27.5 kDa) has been described in rats [5] and Fisher et al [6] suggested that absorption of even larger molecules may be possible. The findings in rodent models have ultimately failed to translate into clinical success largely due to the relatively diminutive presence and distribution of the human olfactory mucosa [7]. However, confirmation of the permeability of nasal mucosa to very large and polar molecules suggests the potential for a novel alternative method.

**Figure 1:**
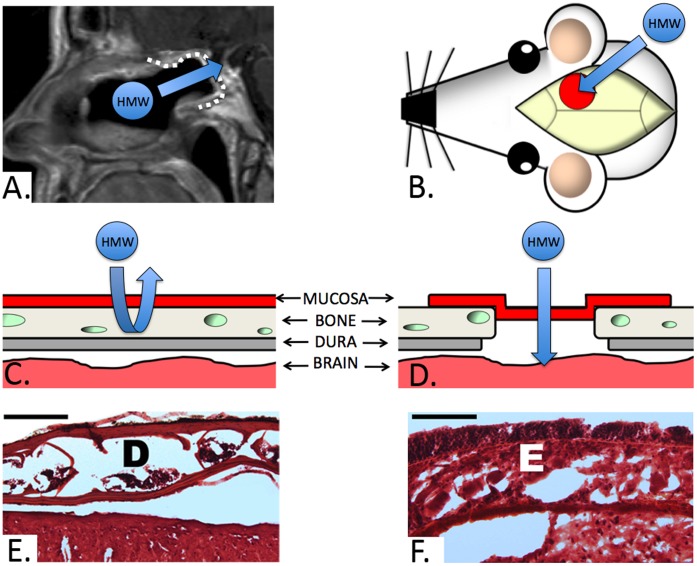
Murine graft model. **A)** Sagittal MRI of a patient following endoscopic reconstruction of a skull base defect using a nasal mucosa graft(dotted white line, arrow denotes the proposed transmucosal pathway for HMW agents from the nose into the CNS through the graft). **B)** Illlustration of the murine graft model with the position of the graft(red circle) relative to the skull. The arrow denotes the equivalent transmucosal pathway to that seen on the MRI(Fig. 1A) utilized in our study. **C and D)** Cross sectional illustration of the skull base layers prior to and following craniotomy with dural removal and mucosal graft inset, respectively. Note that the dural layer(dura and arachnoid) contains the blood-cerebrospinal fluid barrier which restricts the transport of HMW molecules. **E)** Hematoxylin and eosin(H&E) section of the intact murine parietal bone with typical appearance of the inner and outer cortical tables with their associated diploic space(D) prior to engrafting(bar = 200 µm). **F**) H&E section of the mucosal graft implant in direct continuity with underlying brain parenchyma. Note the intact epithelial layer(E) consisting of pseudostratified columnar epithelium.

Using recently developed endoscopic techniques, surgeons are now routinely able to remove brain tumors through the nose without any facial incisions. These approaches require the removal of the intervening dura mater and arachnoid membrane thereby creating a large communication between the interior of the nose and the brain surface. In order to prevent post-operative infection or leakage of cerebrospinal fluid, these holes in the skull base are then sealed using nasal mucosal grafts harvested from the nasal septum [8](Fig. 1A). While these repairs are permanent, water tight, and immunocompetent [9], they also function to replace large regions of the restrictive BCSFB within the arachnoid with relatively permeable sinonasal mucosa. The engrafted nasal mucosa may be subsequently dosed with a variety of therapeutic agents applied topically. Given the lack of underlying arachnoid membrane, these mucosal grafts have the potential to bypass the BBB and transmit HMW or polar agents directly to the brain and subarachnoid space using purely autologous tissues.

The purpose of this study is to determine the capacity of these septal mucosal grafts for permitting diffusion of high molecular weight markers into the CNS. In order to analyze the impact of size and exposure duration on marker delivery, we developed a novel murine extracranial graft model that precisely recapitulates the anatomy and graft morphology encountered at the anterior skull base. Here we provide a proof of concept demonstration that mucosal engrafting of the BBB enables the delivery of HMW molecules to the CNS while obviating the need to implant foreign bodies or penetrate the brain parenchyma.

## Results

### Outcomes of Mucosal Engrafting and Impact on Rhodamine-dextran Marker Uptake

**Figure 2:**
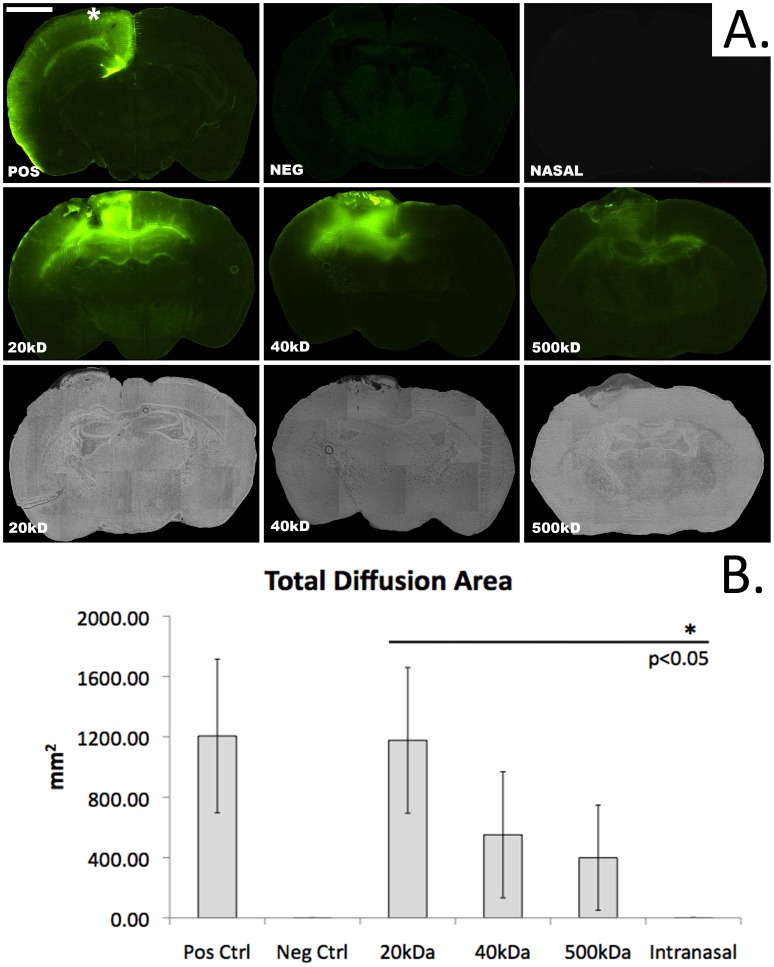
Transmucosal marker diffusion by molecular weight. A) Fluorescent microscopic images demonstrating transmucosal rhodamine-dextran delivery(bar = 1 mm, bregma −1.06 mm, * denotes the position of the reservoir containing the rhodamine-dextran solution in all of the topical delivery conditions). Negative(neg) and positive(pos) control images represent delivery through intact dura or direct parenchymal delivery with no intervening dura or mucosa, respectively. Note the lack of apparent fluorescence in the nasal group in which the 20 kDa rhodamine-dextran solution is delivered directly into the nares. A molecular weight dependent successive reduction in area stained is seen between 20, 40, and 500 kDa rhodamine-dextran. The bottom row represents matched brightfield images demonstrating the mucosal graft stained with Evans blue used to confirm mucosal graft integrity following dosing. B) Graph depicting total area of fluorescent pixels at bregma −1.06 mm following 72 hours of rhodamine-dextran delivery. The total area of delivery under all molecular weight conditions is significantly greater than that seen following direct intranasal instillation of a 20 kDa rhodamine-dextran solution(p<0.05).

To directly examine the efficiency of nasal mucosa in transporting HMW agents, we first developed and validated a murine model to mimic the human skull base. In this model, a mucosal graft harvested from a donor mouse septum was applied over a right parietal craniotomy 3 mm in diameter following dural reflection(Fig. 1B–D). A reservoir was surgically implanted over the mucosal graft to form a tight seal allowing topical dosing of the graft with different fluorescent rhodamine-dextran markers ranging from 20–500 kDa. The mucosal implant procedure was well tolerated and no evidence of subcutaneous infection, mucocele, or distress related to the surgical site was observed during the postoperative period. Hematoxylin and eosin staining demonstrated intact grafts with complete coverage of the bony craniotomy(Fig. 1E,F). Evans blue(EB) staining was used to confirm the integrity of the epithelial tight junctions within the graft following exposure to the rhodamine-dextran solution. The EB dye was restricted to the apical surface in all grafts indicating viable and intact epithelium(Fig. 2A). The validity of the technique was verified by calculating the total area of rhodamine diffusion in the control conditions directly adjacent to the graft(bregma −1.06 mm). The positive control consisted of direct exposure of the brain to the rhodamine-dextran solution with no intervening dura or mucosa. This resulted in a large area of ipsilateral 500 kDa rhodamine-dextran diffusion(1206.03+/−509.44 mm^2^, mean+/−S.D., n = 3). The negative control condition in which the dura was kept intact demonstrated negligible diffusion(1.36+/−1.3 mm^2^, n = 3)(Fig. 2A). Direct intranasal dosing has also been reported to allow for high molecular weight drug delivery to the brain in rodents [5] however this method is inefficient and likely not translatable to humans. In order to directly compare delivery via the mucosal graft with intranasal dosing we applied an equivalent volume of the 20 kDa rhodamine-dextran solution directly into the nostril. The intranasal delivery condition demonstrated minimal delivery which was similar to that of the negative control(1.76+/−2.17 mm^2^, n = 3)(Fig. 2A).

### Transmucosal Rhodamine-dextran Diffusion by Molecular Weight and Time

**Figure 3:**
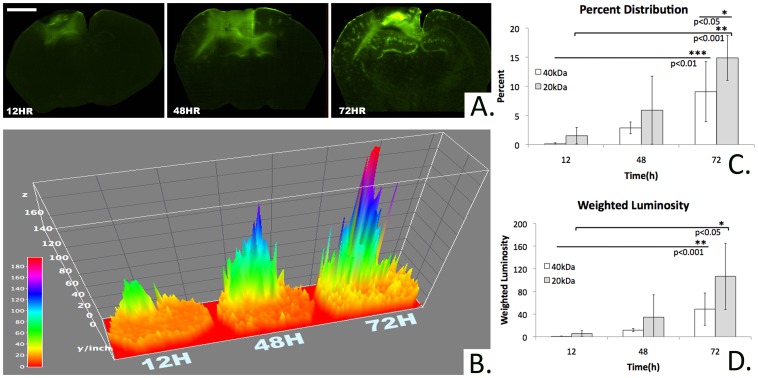
Area and intensity of marker diffusion over time. A)Fluorescent microscopic images demonstrating an increase in area and intensity of transmucosal rhodamine-dextran delivery over time(bar = 1 mm, bregma −1.06 mm, 40 kDa rhodamine-dextran). B) 3-D map of Fig. 3A quantifying increase in relative pixel luminosity intensity across each cross section over time(bregma −1.06 mm, 40 kDa rhodamine-dextran). C) Percent of the total cross sectional area containing detectable rhodamine fluorescence at bregma −1.06 mm. The overall trend describes an increasing percent area of staining as time increases and molecular weight decreases. Among the 40 kDa conditions, the percent area of rhodamine staining at 72 h is significantly greater than at 12 or 48 h. Among the 20 kDa conditions the percent area of rhodamine staining at 72 h is significantly greater than at 12 h. At 72 h, the percent area subtended by the 20 kDa condition is significantly greater than that of the 40 kDa condition. D) Weighted luminosity of rhodamine staining at bregma −1.06 mm. The overall trend describes an increasing weighted luminosity as time increases and molecular weight decreases. Among the 40 kDa conditions, the weighted luminosity at 72 h is significantly greater than at 12 or 48 h. Among the 20 kDa conditions, the weighted luminosity at 72 h is significantly greater than at 12 h.

We next examined the impact of molecular weight and duration of exposure on transmucosal rhodamine-dextran diffusion. Polymers of molecular weights 20, 40 and 500 kDa were tested. At designated time points, mice were euthanized and coronal brain slices were imaged in order to calculate the area and intensity of rhodamine within a cross section incorporating the leading edge of the mucosal graft. As shown in Fig. 2A the degree of marker diffusion at 24 hours was directly related to polymer molecular weight. The calculated area of polymer diffusion after 72 hours of administration shows a trend of an increase in area with decreasing molecular weight (Fig. 2B). We next focused on the distribution of the more therapeutically relevant lower molecular weight markers. The 20 kDa marker demonstrated a trend towards greater percent distribution and weighted luminosity than the 40 kDa dextran at all time points. This trend reached statistical significance for the percent distribution at 72 h(p<0.05)(Fig. 3).

The percent of total cross sectional area stained by rhodamine successively increased with longer exposure(Fig. 3A,B). The 72 h time point demonstrated the greatest percent distribution for both the 20 and 40 kDa dextran markers(14.89+/−3.86%, 9.08+/−5.16%, mean+/−S.D.; n = 3, respectively)(Fig. 3C). In order to further compare the relative concentrations of rhodamine delivery over time, a weighted luminosity average was calculated over the entire cross section. For both the 20 and 40 kDa dextran molecules, the weighted luminosity successively increased over time with the 72 h time point demonstrating the greatest intensity(106.46+/−58.39, 48.65+/−28.46; n = 3, respectively)(Fig. 3D).

### Striatal Delivery of Transmucosal Rhodamine-dextran

**Figure 4:**
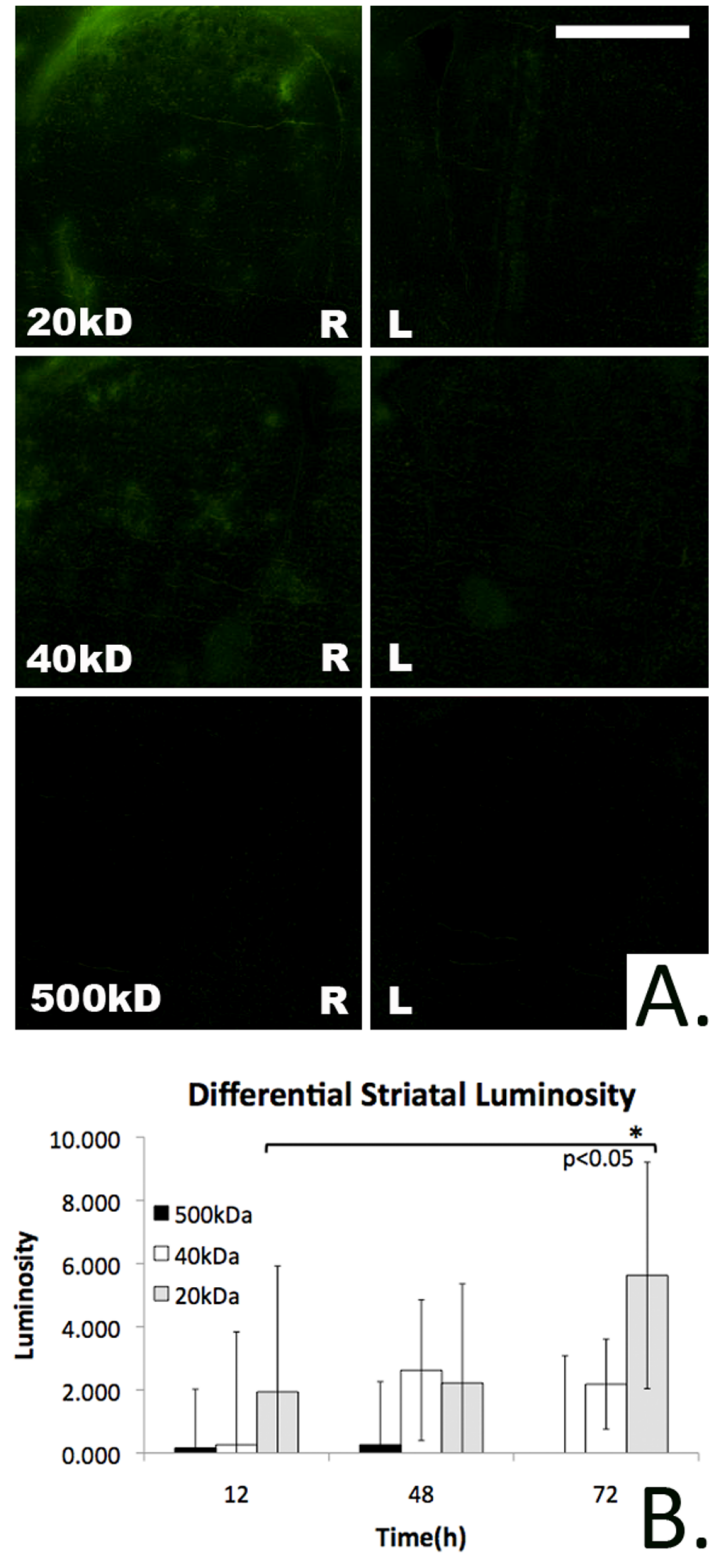
Transmucosal marker delivery to striatum over time. A) Fluorescent microscopic images demonstrating transmucosal rhodamine-dextran delivery to the striatum(bregma 1.18 mm, bar = 0.5 mm, 72 h). Diffusion into the right striatum(ipsilateral to mucosal graft) occurs in a molecular weight dependent fashion while contralateral diffusion to the left striatum is negligible. B) The differential luminosity of the right(ipsilateral) striatum relative to the contralateral striatum following rhodamine-dextran exposure. While detectable striatal diffusion of the 20 and 40 kDa rhodamine-dextran conditions is seen, the 500 kDa rhodamine-dextran solution is associated with negligible striatal delivery. The differential luminosity of the striatum at 72 h following 20 kDa rhodamine-dextran delivery is significantly greater than at 12 h.

In order to ascertain whether the mucosal graft method could be utilized to deliver macromolecular therapies to the striatum for treating Parkinson’s disease(PD), striatal delivery was examined. The difference between the average luminosity of the right(ipsilateral to the mucosal graft) and left striatum(bregma 1.18 mm) was calculated over 12 to 72 hours of continuous exposure to the 20, 40, and 500 kDa rhodamine-dextran marker solutions. Ipsilateral striatal staining was seen among both the 20 and 40 kDa conditions however staining was negligible for the highest molecular weight 500 kDa polymer. The differential striatal luminosity at 72 hours for the 20 kDa marker was significantly greater than that at 12 hours(5.62+/−3.58 vs. 1.94+/−3.99, mean+/−S.D., n = 3, p<0.05)(Fig. 4). As expected, contralateral staining was not detected for any of the molecular weights or exposure durations. These results demonstrate the feasibility of delivering HMW therapies such as growth factors to the striatum as these proteins are of comparable molecular weights to the polymers tested in this study. Among all conditions no significant delivery to the substantia nigra was evident.

## Discussion

The BBB represents one of the major obstacles to the development and implementation of therapies for the treatment of neurodegenerative disease. Neurotrophic factors with known disease modifying capabilities in Parkinson’s Disease have failed to be adopted into routine clinical use due to, in part, their inability to cross the BBB. Glial derived neurotrophic factor(GDNF) represents one such molecule which can promote mesencephalic dopaminergic neuronal survival and modulate neuronal function [10]. Given the size of the dimeric GDNF peptide(25–30 kDa), therapeutic efficacy studies have required the implantation of intracerebroventricular or intraputamenal catheters in order to provide access to the CNS. One clinical trial examining direct intraputamenal delivery reported that all patients developed vasogenic edema at the catheter tip and 40% required additional catheter manipulation or explantation [11]. These findings suggest that while direct, continuous delivery of neurotrophic or other experimental HMW molecules to the CSF have tremendous potential, there remains a need for a safer and more permanent alternative to the transcranial implantation of indwelling foreign bodies.

Intranasal drug delivery has been investigated as an alternative potential method to deliver therapeutic agents to the CNS thereby avoiding trauma to the brain parenchyma or implantation of foreign bodies. While the intranasal route for systemic administration is currently utilized for PD therapies such as apomorphine [12], its utility in direct CNS delivery is controversial. The anatomic basis for this theory arises from the fact that the subarachnoid space extends into the nasal cavity along the olfactory neurons allowing for the rapid uptake of large molecules into the CSF compartment [13]. While this delivery route has been successfully demonstrated in animal models [14], its clinical utility is less clear. The relative paucity of human olfactory mucosa coupled with its location in a region with little access for topical agents [15] suggests that these rodent studies may vastly overstate the clinical potential of transnasal CNS delivery through an intact skull base. In a critical analysis of 100 papers examining this pathway only 2 approached satisfactory evidence for utility of the transnasal route [16]. Our findings demonstrating a relative lack of rhodamine uptake following intranasal instillation supports this contention.

The described mucosal grafting method represents an adaptation of a surgical technique which is currently in widespread use in the field of endoscopic skull base surgery and is, in fact, considered the gold standard in reconstruction of skull base defects [9], [17]. In order to test the feasibility of direct transmucosal CNS drug delivery, an appropriate animal model had to be developed and validated. While the described murine extracranial model does not replicate the intranasal milieu, it precisely recapitulates the skull base morphology following surgical mucosal graft reconstruction. The discreet lack of rhodamine-dextran uptake in the negative control condition confirmed that the BCSFB was present and intact in the murine arachnoid. Additionally it was critical to validate the integrity of each mucosal graft following rhodamine-dextran exposure to ensure that the observed absorption could not be attributed to a disruption of the epithelium secondary to poor healing or surgical trauma. The described Evans blue testing confirmed that all of the observed rhodamine-dextran absorption resulted from transport through an intact mucosal graft.

Our data demonstrate that while the mucosal grafts were permeable to all of the dextran molecules tested, there was a clear and significant decrement in transport as molecular weight increased. This supports that fact that the mucosal epithelium, while significantly more permeable than the BBB, also exerts some measure of size selectivity on the molecules it is exposed to. This was overcome, in part, by extending the exposure duration. This could be achieved clinically using a variety of drug eluting polymers. Given the potential for use of this technique for neurotrophic factor delivery in PD, this study also sought to examine the relative uptake in the striatum, a potential end organ target for GDNF. Our data show that while the 500 kDa could be detected, significant ipsilateral delivery was seen only up to 40 kDa. The lack of contralateral diffusion is consistent with the unilateral bias of the mucosal graft coupled with the known limitations of drug diffusion through the extracellular spaces within the brain [18]. In clinical use however, the mucosal graft is positioned in the midline proximal to the mesencephalon and adjacent to the prepontine and interpeduncular cisterns thereby providing bilateral access for drug delivery.

One limitation of this study is that it does not address whether the findings in our animal model would be applicable in a clinical setting due to regional differences in convection throughout the brain. When directly comparing the murine model to the human skull base however, the reported findings may actually underestimate the potential for drug delivery for two important reasons. The first is that in the human, the relative mucosal graft area to brain volume ratio would be much higher given that grafts up to 20cm^2^ are achievable. Additionally in the clinical scenario, diffusion to more distal aspects of the brain would be possible via CSF circulation, a finding not seen in the murine model due to occlusion of the smaller subarachnoid space following the craniotomy.

The intranasal mucosal engrafting technique has the potential to bypass the BBB providing a permanent route for direct CNS drug delivery using only autologous tissues. The mucosal graft represents a non-specific semipermeable membrane that could be compatible with both established and experimental HMW therapeutics including neurotrophic factors, fusion proteins [10], nanoparticles [19], and viruses [20]. This proof of concept study also suggests that the mucosal graft enables the delivery of molecules up to 40 kDa(the size of GDNF) to the striatum while obviating the need to implant foreign bodies or penetrate the brain parenchyma. Given the fact that this surgical technique is currently in clinical use, additional efforts will be directed at therapeutic efficacy studies which will form the basis for future clinical trials.

## Materials and Methods

### Ethics

All procedures involving animals are approved by the Boston University Institutional Animal Care and Use Committee.

### Experimental Design

Following engrafting of the heterotopic mucosal graft(described under surgical methods) in 20–25 g C57BL/6 mice, the mucosa is exposed to a 60 µL rhodamine-dextran solution. The administered solutions are standardized by fluorophore content. Rhodamine conjugated dextran solutions (Sigma, molecular weights of 20, 40, or 500 kDa) were dissolved in saline and titrated until an absorbance of 0.67 at 520 nm is obtained. Absorbance spectra are obtained using 2 µL of solution in a NanoDrop 2000 spectrophotometer(Thermo Scientific). Positive control groups are mice in which the rhodamine-dextran solution is applied directly to the parenchymal surface following craniotomy and reflection of the underlying dura and arachnoid membrane. Negative control groups are mice in which a craniotomy is performed leaving the underlying dura and arachnoid intact. The intranasal group consists of direct instillation of 60 µL of the 20 kDa rhodamine-dextran solution in 10 µL increments while the mouse was anesthetized using isoflurane.

### Surgical Methods

In order to perform the heterotopic mucosal engrafting procedure, a donor mouse is used to harvest the nasal septum *en bloc* according to previously described methods [21]. The septal mucoperichondrial flaps are preserved in normal saline for no more than 2 hours prior to engrafting. The experimental mouse is anesthetized using isoflurane and placed in a stereotaxic frame below an operating microscope. Following standard prepping and clipping of the scalp, a linear sagittal incision is made from the level of the mid orbit to the occiput. Flaps are elevated bilaterally in a subgaleal plane exposing the pericranium. The pericranium is then cleared from the intended craniotomy site centered at −1.5 mm anterior-posterior, and∼2 mm medial-lateral to bregma. Using a drill, a craniotomy 3 mm in diameter is outlined. The bone is then carefully reflected leaving the underlying dura intact. In the experimental mice, the dura and arachnoid are then reflected leaving the underlying pia mater undisrupted. This is accomplished by applying Vetbond(3 M) to the dural surface. Once dried, the membrane can be manually removed without damaging the brain surface. The previously harvested mucosal graft is then inset taking care to place the mucoperichondrial layer against the exposed pia mater. The skin flaps are then closed and the flap is left to engraft for 7 days.

On post-surgical day 7 the skin flaps are reflected taking care to leave the underlying mucosal graft undisturbed. The graft is then inspected using a dissecting microscope to ensure there is no evidence of defects, infection, necrosis, or exposed bony edges. A 100 µL polypropylene reservoir is then inset over the graft and secured to the cranium using polycyanoacrylate followed by dental cement(Stoelting). The reservoir is checked for leaks and the desired rhodamine-dextran solution is then applied to the reservoir ensuring the solution comes into direct contact with the mucosal surface.

### Histologic Preparation and Imaging

Following rhodamine-dextran dosing according to the assigned group, the solution is removed and replaced with Evans blue(EB, 961Da, Sigma) for 1 hour. Defects or disruptions in the graft epithelium will allow for EB diffusion into the underlying brain parenchyma over this short exposure time. Apical restriction of the dye is therefore used to confirm graft integrity during the preceding rhodamine-dextran dosing interval. The mice are euthanized using pentobarbital until unresponsive to a foot withdrawal reflex. The brain is then harvested *en bloc* and snap frozen in a 2-methylbutane dry ice bath. The brain is then mounted in OCT(Sigma) and sectioned at a thickness of 50 µm using a crytostat(Leica). In order to prevent rhodamine diffusion into the mounting media, sections are immediately imaged using an Olympus IX81 microscope equipped with Metamorph software. Rhodamine containing samples are imaged at 4× using a red filter with a 1000 ms exposure time. Evan’s Blue containing samples are imaged at 4× in phase-contrast mode with a 15 ms exposure time. Images are merged and exported for luminosity analysis in tagged image file format(TIFF).

### Image Analysis

Rhodamine luminosity is quantified according to methods adapted from Kirkeby et al [22]. All TIFF files are imported into a graphics editing program(Adobe Photoshop) for analysis which was confirmed using Image J. The brightness across all images is normalized using a non-tissue bearing region of the slide. The software is used to automatically select pixels exceeding the background luminosity tolerance threshold in each image. The total number of pixels and mean luminosity of all selected pixels are recorded for each image. A weighted luminosity was calculated by multiplying the average luminosity over all pixels by the total number of fluorescent pixels. In order to measure striatal luminosity, a brightfield image is superimposed over the fluorescent image and the striatum was selected by hand. The brightfield image is then removed and the luminosity of the selected region is recorded. The differential luminosity is calculated by subtracting the luminosity of the ipsilateral from the contralateral striatum(relative to the graft).

### Statistics

The significance of differences across the experimental and control conditions is determined using a 2 tailed Student’s t-test(SigmaStat v3.1, Systat Software Inc, San Jose, CA). Differences are considered statistically significant at p<0.05.
